# The Distinct Performances of Ultrasound, Mammograms, and MRI in Detecting Breast Cancer in Patients With Germline Pathogenic Variants in Cancer Predisposition Genes

**DOI:** 10.3389/fonc.2021.710156

**Published:** 2021-07-13

**Authors:** Jiaqi Liu, Xin Wang, Lin Dong, Xin Huang, Hengqiang Zhao, Jiaxin Li, Shengkai Huang, Pei Yuan, Wenyan Wang, Jie Wang, Zeyu Xing, Ziqi Jia, Yue Ming, Xiao Li, Ling Qin, Gang Liu, Jiang Wu, Yiqun Li, Menglu Zhang, Kexin Feng, Jianming Ying, Xiang Wang

**Affiliations:** ^1^ Department of Breast Surgical Oncology, National Cancer Center/National Clinical Research Center for Cancer/Cancer Hospital, Chinese Academy of Medical Sciences and Peking Union Medical College, Beijing, China; ^2^ Department of Pathology, National Cancer Center/National Clinical Research Center for Cancer/Cancer Hospital, Chinese Academy of Medical Sciences and Peking Union Medical College, Beijing, China; ^3^ Department of Breast Surgery, Peking Union Medical College Hospital, Peking Union Medical College and Chinese Academy of Medical Sciences, Beijing, China; ^4^ Department of Orthopedic Surgery, Key Laboratory of Big Data for Spinal Deformities, Beijing Key Laboratory for Genetic Research of Skeletal Deformity, Peking Union Medical College Hospital, Peking Union Medical College and Chinese Academy of Medical Sciences, Beijing, China; ^5^ Department of Laboratory Medicine, National Cancer Center/National Clinical Research Center for Cancer/Cancer Hospital, Chinese Academy of Medical Sciences and Peking Union Medical College, Beijing, China; ^6^ Department of Breast Surgery, Beijing Tiantan Hospital, Capital Medical University, Beijing, China; ^7^ Department of Ultrasound, National Cancer Center/National Clinical Research Center for Cancer/Cancer Hospital, Chinese Academy of Medical Sciences and Peking Union Medical College, Beijing, China; ^8^ PET-CT Center, National Cancer Center/National Clinical Research Center for Cancer/Cancer Hospital, Chinese Academy of Medical Sciences and Peking Union Medical College, Beijing, China; ^9^ Department of Radiology, Peking Union Medical College Hospital, Peking Union Medical College and Chinese Academy of Medical Sciences, Beijing, China; ^10^ Department of Breast Surgical Oncology, Cancer Hospital of HuanXing, Beijing, China; ^11^ Department of Oncology, National Cancer Center/National Clinical Research Center for Cancer/Cancer Hospital, Chinese Academy of Medical Sciences and Peking Union Medical College, Beijing, China

**Keywords:** hereditary breast cancer, *BRCA1/2*, mammography, ultrasonography, magnetic resonance imaging

## Abstract

A proportion of up to 10% of breast cancer resulted from hereditary germline pathogenic variants (GPVs) in cancer predisposition genes (CPGs), which been demonstrated distinct clinical features and imaging manifestations. However, the performance of imaging modalities for breast cancer surveillance in CPG mutation-carriers is still unclear, especially in Asian women. A population of 3002 breast cancer patients who received germline genetic testing of CPGs was enrolled from three hospitals in China. In total, 343 (11.6%) patients were found to harbor GPVs in CPGs, including 137 (4.6%) in *BRCA1* and 135 (4.6%) in *BRCA2*. We compared the performances of ultrasound, mammograms, MRI, and the combining strategies in CPG mutation carriers and non-carriers. As a result, the ultrasound showed a higher detection rate compared with mammograms regardless of the mutation status. However, its detection rate was lower in CPG mutation carriers than in non-carriers (93.2% *vs* 98.0%, *P*=2.1×10^-4^), especially in the *BRCA1* mutation carriers (90.9% *vs* 98.0%, *P*=2.0×10^-4^). MRI presented the highest sensitivity (98.5%) and the lowest underestimation rate (14.5%) in CPG mutation carriers among ultrasound, mammograms, and their combination. Supplemental ultrasound or mammograms would add no significant value to MRI for detecting breast cancer (*P*>0.05). In multivariate logistic regression analysis, the family or personal cancer history could not replace the mutation status as the impact factor for the false-negative result and underestimation. In summary, clinicians and radiologists should be aware of the atypical imaging presentation of breast cancer in patients with GPVs in CPGs.

## Introduction

Breast cancer is currently the most common cancer among women both in the West and East (1, 2). A proportion of 5-10% breast cancer resulted from hereditary germline pathogenic variants (GPVs) in cancer predisposition genes (CPGs) such as *BRCA1/2*, *PALB2*, etc. (3–5) The BRCA-related breast cancer has demonstrated distinct clinical phenotypes in pathology features and imaging manifestations (6). Thus, special breast cancer screening and diagnosis guidelines with higher sensitivity have been applied in the CPG mutation-carriers in the US and UK (7, 8). However, the performance of imaging modalities in detecting BC in Asian CPG mutation carriers was still unknown.

The mammogram alone is insufficient for young women carrying *BRCA1/2* mutations, even in women with low breast density (9). Compared to mammograms, the dynamic contrast-enhanced breast magnetic resonance imaging (MRI) has demonstrated the highest sensitivity in *BRCA1/2* mutation-carriers (10, 11). Thus, the National Comprehensive Cancer Network (NCCN) has recommended annual breast MRI combined with an annual mammogram in breast cancer surveillance for women with *BRCA1/2* mutations (7); while both the United States Preventive Services Taskforce (USPSTF) and the WHO International Agency for Research on Cancer (IARC) do not provide clear screening recommendations (12). Considering the high cost and high false-positive rate of the MRI, ultrasonography is widely used as a supplemental screening modality in Asian countries (13). It has also significantly increased the detection rate and screening sensitivity (14). A recent meta-analysis of 21 studies showed that supplemental ultrasound shows added value to sensitivity in women with dense breasts compared with mammograms alone (15). However, the clinical utility of ultrasound for detecting breast cancer in CPG mutation-carriers remains unclear (16).

Here, we investigated whether the germline variants could impact the performance of the mammogram, ultrasound, and MRI in a multi-center cohort of 3002 female Chinese breast cancer patients undergoing the multigene testing. This is also the first study to investigate the diagnosis accuracy and the effectiveness of these imaging techniques in screening for breast cancer among Chinese women with CPG mutations.

## Methods

### Study Participants and Design

This multicenter cohort study recruited consecutive female patients with breast cancer from October 1, 2017, to July 31, 2020, at the Cancer Hospital and Peking Union Medical College Hospital, both of Chinese Academy of Medical Sciences and Peking Union Medical College, and Huanxing Cancer Hospital, all in Beijing, China. Ultrasonography was conducted as the screening modality for all the patients. Digital mammography was provided for patients who were suspected for calcification in the breast or older than 40 years old. The screening MRI was performed according to patients’ willingness. The diagnosis of each patient was based on the pathology results from resection specimens. This study followed the Strengthening the Reporting of Observational Studies in Epidemiology (STROBE) reporting guideline (17).

### Clinical Evaluation

We collected phenotypic data including the onset age, family history, personal cancer history, imaging evaluation, pathology features, clinical subtype, and clinical stage. Clinical grouping of subtypes was defined by the status of hormone receptor and HER2 according to the St. Gallen 2017 criteria (18). Standard digital mammography, ultrasonography, and MRI techniques were conducted at each center. The images were interpreted and classified according to the fifth edition of the Breast Imaging Reporting and Data System (BI-RADS) standard by two experienced radiologists independently at each center blind to the mutation status and the pathological finding (19). The BI-RADS 0 findings were excluded in the further analysis.

### Germline Variants Analysis

Genomic DNA was extracted from peripheral blood or saliva. Germline variants were analyzed by a multiplex amplicon-based library preparation system and targeted a panel covering the coding regions and consensus splice sites of 50 CPGs in DNA-repair pathways for sequencing using an Illumina HiSeq 4000 Platform (20). The cancer predisposition genes included *ATM, BARD1, BRIP1, BRCA1, BRCA2, CDH1, PALB2, RAD5IC, RAD51D, CHEK2, NBN, TP53, PTEN, STKI1, APC, MUTYH, MLH1, MSH2, MSH6, PMS2, SMAD4, KIT, PDGFA, HOXB13, RB1, PTCH1, CDK4, CDKN2A, PALLD, WRN, MEN1, RECQL, RET, SDHA, SDHB, SDHC, SDHD, SDHAF2, GNAS, MAX, VHL, MET, FH, FLCN, TSC1, TSC2, PRKAR1A, SMARCA4, SMARCB1*, and *BRAF*. The clinical significance (benign/likely benign/variant of unknown significance/likely pathogenic/pathogenic) of each variant was annotated according to the ACMG/AMP guidelines (21). Pathogenic and likely pathogenic variants were analyzed together as pathogenic variants. Benign and likely benign variants were analyzed together as benign variants. The mutation curation was also conducted by two experienced medical geneticists independently blind to the imaging interpretation.

### Statistical Analysis

The false-negative rate (FNR) was defined as the proportion of the BI-RADS categories less than 4 (22). The underestimation rate (UR) was defined as the proportion of the estimated malignancy rate of less than 50% (the BI-RADS categories 0-4b). The Student’s t-test was used to analyze the onset age and tumor size. The prevalence of the lymph nodes metastasis, FNRs, and URs were compared using the Pearson χ2 test or the Fisher’s exact test to obtain *p* values and odds ratios (ORs) with 95% confidence intervals (CIs). We also conducted multivariate logistic regression to evaluate the impact of the characteristics on the diagnostic sensitivity of different imaging techniques. Statistical tests were two-sided, and *p* values <0.05 were considered significant. Two-side *p*<0.05 was considered as statistically significant. Statistical analysis was performed using SPSS version 15.0 (SPSS, USA) and R statistical software, version 3.5.1.

## Results

### Patient Characteristics

**Figure 1:**
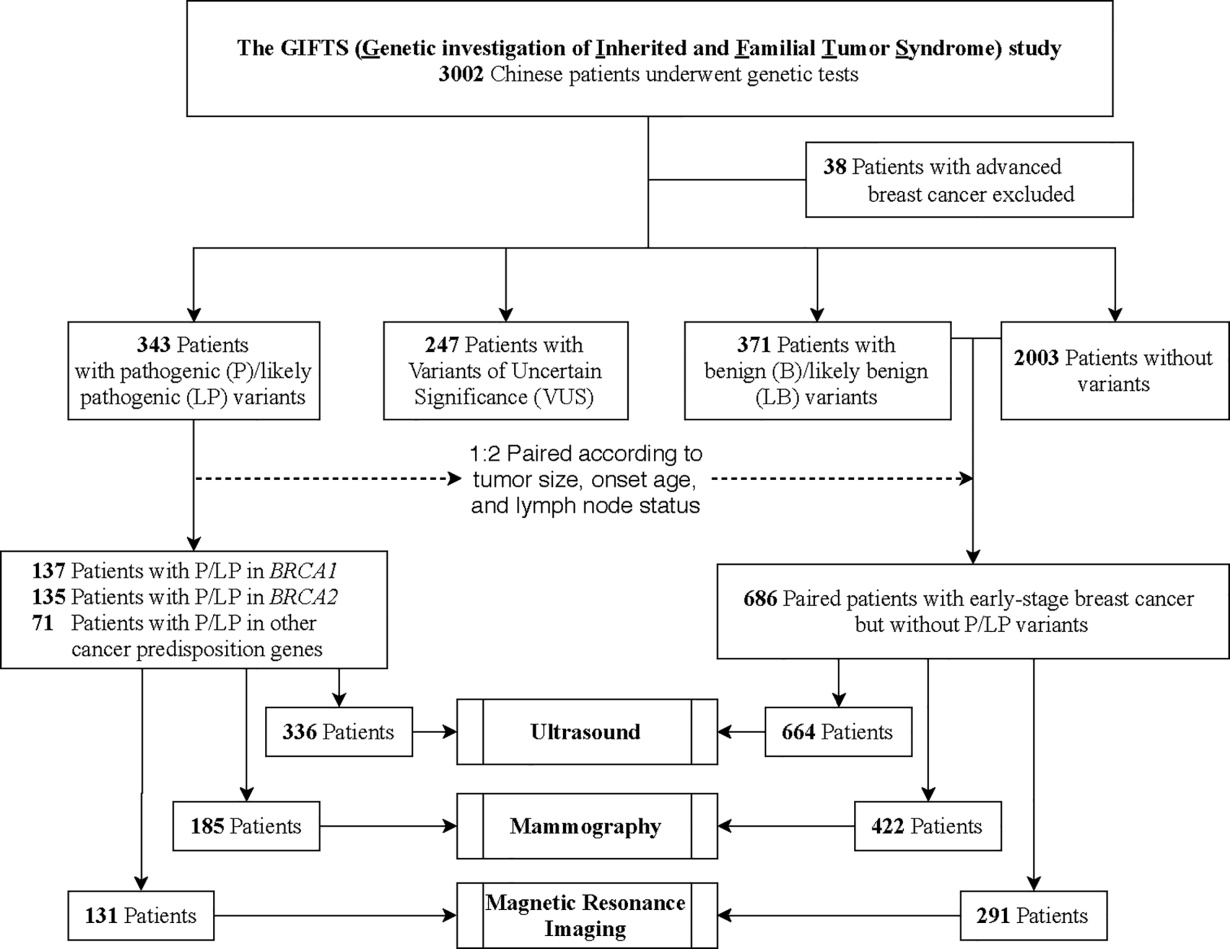
Patient Enrollment and Study Design. In this study, 3002 breast cancer patients were enrolled from three hospitals in China. Thirty-eight patients with advanced breast cancer were excluded. In total, 343 patients were found to harbor germline pathogenic variants (GPVs) in cancer predisposition genes (CPGs). To compare performances of ultrasound, mammograms, Magnetic Resonance Imaging (MRI), and combining strategies between different mutation status, 686 non-carriers were selected as 1:2 paired with the CPG mutation-carriers according to the onset age, tumor size, and lymph node status.

In this study, 3002 women who were diagnosed with breast cancer were enrolled from three hospitals in China at a mean ± SD age of 42.8 ± 9.0 years (**Figure 1**). Thirty-eight patients with advanced breast cancer were excluded. In total, 343 (11.6%, 343/2964) patients were found to harbor GPVs in CPGs, including 137 (4.6%) in *BRCA1*, 135 (4.6%) in *BRCA2*, and 71 (2.4%) in other CPGs. Besides, 247 patients with variants of uncertain significance were excluded from further analysis (**Figure 1**).

The age of diagnosis is significantly younger in patients with GPVs as compared with patients without GPVs (40.3 ± 7.9 *vs*. 43.3 ± 9.3, respectively, *p*=1.4×10^-8^), and even younger in patients with GPVs of *BRCA1* (39.1 ± 7.7 *vs*. 43.3 ± 9.3, *p*=1.5×10^-7^; **Table 1**). For pathological characteristics, there was no association between the tumor size and mutation status (*p*=0.93). A higher proportion of invasive ductal carcinoma was identified in patients with GPVs in *BRCA1/2* (94.9% and 86.7% in *BRCA1* and *BRCA2* mutation-carriers *vs*. 77.9% in non-carriers, *p*=1.3×10^-7^ and 0.02), while less ductal carcinoma *in situ* (DCIS) was found in patients with GPVs in *BRCA1* (0% in *BRCA1* mutation-carriers *vs*. 5.5% in non-carriers, *p*=1.1×10^-3^). In patients with GPVs in *BRCA1*, there was less proportion of histological grade I and II than patients without GPVs (0% in grade I and 19.7% in grade II in *BRCA1* mutation-carriers *vs*. 5.8% and 42.8% in non-carriers, *p*=6.9×10^-4^ and 3.4×10^-8^, respectively), but a higher proportion of grade III (73.0% in *BRCA1* mutation-carriers *vs*. 25.7% in non-carriers, *p*=4.9×10^-29^). Compared to the patients without GPVs, less grade I in patients with PGVs in *BRCA2* (5.8% *vs*. 1.5%, *p*=0.03), while more grade II in patients with PGVs in *BRCA2* and other CPGs were identified (42.8% in non-carriers *vs*. 52.6% in *BRCA2* mutation-carriers and 56.3% in other CPG mutation-carriers, both *p*=0.03, respectively).

**Table 1:** Comparison of clinical and pathological characteristics between patients with and without cancer predisposition gene mutations.

Clinical characteristics	Without GPVs (n = 2374)	All CPG mutation-carriers (n = 343)	*BRCA1* mutation-carriers (n = 137)	*BRCA2* mutation-carriers (n = 135)	Other CPG mutation-carriers (n = 71)	*P* 1^a^	*P* 2	*P* 3	*P* 4
Age of onset^b^	43.3 ± 9.3	40.3 ± 7.9	39.1 ± 7.7	41.4 ± 8.0	40.7 ± 7.9	**1.4×10^-8^**	**1.5×10^-7^**	**0.02**	**0.02**
Tumor size^b^	2.2 ± 1.3	2.2 ± 1.1	2.3 ± 1.1	2.3 ± 1.1	2.0 ± 1.1	0.93	0.59	0.65	0.22
Histology^c^									
IDC	1849 (77.9%)	308 (89.8%)	130 (94.9%)	117 (86.7%)	61 (85.9%)	**6.7×10^-8^**	**1.3×10^-7^**	**0.02**	0.14
DCIS	131 (5.5%)	6 (1.7%)	0 (0%)	4 (3.0%)	2 (2.8%)	**1.4×10^-3^**	**1.1×10^-3^**	0.24	0.43
Grade^c^									
I	137 (5.8%)	3 (0.9%)	0 (0%)	2 (1.5%)	1 (1.4%)	**1.1×10^-5^**	**6.9×10^-4^**	**0.03**	0.18
II	1017 (42.8%)	138 (40.2%)	27 (19.7%)	71 (52.6%)	40 (56.3%)	0.38	**3.4×10^-8^**	**0.03**	**0.03**
III	609 (25.7%)	158 (46.1%)	100 (73.0%)	44 (32.6%)	14 (19.7%)	**4.7×10^-14^**	**4.9×10^-29^**	0.09	0.33
Clinical subtype^c^									
HR+/HER2-	971 (40.9%)	145 (42.3%)	31 (22.6%)	77 (57.0%)	37 (52.1%)	**0.64**	**1.4×10^-5^**	**3.0×10^-4^**	0.07
HR+/HER2+	297 (12.5%)	13 (3.8%)	1 (0.7%)	6 (4.4%)	6 (8.5%)	**1.6×10^-7^**	**8.4×10^-7^**	**3.9×10^-3^**	0.37
HR-/HER2-	353 (14.9%)	135 (39.4%)	94 (68.6%)	22 (16.3%)	19 (26.8%)	**5.1×10^-24^**	**4.7×10^-42^**	0.62	**0.01**
HR-/HER2+	220 (9.3%)	4 (1.2%)	1 (0.7%)	1 (0.7%)	2 (2.8%)	**2.8×10^-9^**	**6.7×10^-5^**	**6.6×10^-5^**	0.06
Lymph nodes status^c^									
Positive	907 (38.2%)	169 (49.3%)	51 (37.2%)	82 (60.7%)	36 (50.7%)	**0.01**	0.28	**1.7×10^-6^**	**0.04**

a P2 Non-carriers vs. BRCA1 mutation-carriers, P3 Non-carriers vs. BRCA2 mutation-carriers, P4 Non-carriers vs. other CPGs mutation-carriers.

b Mean ± SD, yr, student T test.

c No. (%), Pearson’s chi-square test or Fisher’s exact test.

CPG, cancer predisposition genes; MRI, magnetic resonance imaging.

For the molecular subtype, more triple-negative breast cancers were found in patients with GPVs in *BRCA1* and other CPGs than the non-carriers (68.6% and 26.8% *vs*. 14.9%, *p*=4.7×10^-42^ and 0.01; **Table 1**). Significantly fewer HER2 positive breast cancers including both HR-/HER2+ and HR+/HER2+ were found in patients with GPVs in *BRCA1/2* than the non-carriers (**Table 1**). However, there were more HR+/HER2- breast cancers in *BRCA2* mutation-carriers (57.0%) and fewer in *BRCA1* mutation-carriers (22.6%) than the non-carriers (40.9%, *p*=1.4×10^-5^ and 3.0×10^-4^, respectively).

In addition, more patients with lymph node metastasis were found in *BRCA2* and other CPGs subgroups than the non-GPVs group (60.7% in *BRCA2* mutation-carriers and 50.7% in other CPGs mutation-carriers vs. 38.2% in non-carriers, *p*=1.7×10^-6^ and 0.04, respectively).

### The Diagnosis Accuracy of the Breast Imaging Modalities

To compare the performances of imaging modalities between different mutation status, 686 non-carriers were selected as 1:2 paired with the CPG mutation-carriers (n=343) according to the onset age, tumor size, and lymph node status (**Figure 1**). The CPG mutation-carriers were further divided into *BRCA1* mutation-carriers (n=137), *BRCA2* mutation-carriers (n=135), and other CPGs mutation-carriers (n=71) according to the mutated genes (**Table 2**). As all the patients underwent ultrasound, 7 patients with mutations and 22 patients without mutations were diagnosed as BI-RADS 0 category. Therefore, the performance of ultrasound was evaluated in 336 patients with mutations and 664 patients without mutations. Meanwhile, the performance of digital mammography and MRI was evaluated in 185 and 131 patients with mutations and 422 and 291 patients without mutations, respectively.

**Table 2:** The performance of imaging modalities in patients with cancer predisposition gene mutations and pair non-mutation controls.

	Paired non-CPG controls (n = 686)	All CPG mutation -carriers (n = 343)	*BRCA1* mutation-carriers (n = 137)	*BRCA2* mutation-carriers (n = 135)	Other CPG mutation-carriers (n = 71)	*P* 1^a^	*P* 2	*P* 3	*P* 4
**Clinical characteristics**									
Age of onset^b^	40.5 ± 8.2	40.3 ± 7.9	39.1 ± 7.7	41.4 ± 8.0	40.7 ± 7.9	0.70	0.05	0.26	0.88
Tumor size^b^	2.2 ± 1.1	2.2 ± 1.1	2.3 ± 1.1	2.3 ± 1.1	2.0 ± 1.1	0.78	0.68	0.90	0.12
Lymph nodes positive^c^	50.9% (349/686)	49.3% (169/343)	37.2% (51/137)	60.7% (82/135)	50.7% (36/71)	0.64	**3.5×10^-3^**	**0.04**	1.00
**Imaging accuracy**									
Ultrasound									
FNR^d^	2.0% (13/664)	6.8% (23/336)	9.1% (12/132)	4.5% (6/133)	7.0% (5/71)	**2.1×10^-4^**	**2.0×10^-4^**	0.11	**0.02**
UR^e^	18.7% (124/664)	35.4% (119/336)	37.1% (49/132)	33.1% (44/133)	36.6% (26/71)	**1.5×10^-8^**	**9.0×10^-6^**	**4.2×10^-4^**	**9.3×10^-4^**
Δ size^f^	0.0 ± 0.9	0.0 ± 1.1	0.0 ± 1.0	-0.1 ± 1.2	0.3 ± 1.2	0.51	0.76	0.41	0.05
Mammograms									
FNR^d^	21.3% (90/422)	25.4% (47/185)	30.4% (24/79)	20.0% (14/70)	25.0% (9/36)	0.29	0.08	0.88	0.67
UR^e^	44.1% (186/422)	56.2% (104/185)	60.8% (48/79)	52.9% (37/70)	52.8% (19/36)	**6.3×10^-3^**	**6.9×10^-3^**	0.20	0.38
Δ size^f^	0.1 ± 1.1	0.0 ± 1.4	-0.1 ± 1.4	-0.2 ± 1.4	0.5 ± 1.2	0.46	0.30	0.19	0.07
MRI									
FNR^d^	0.7% (2/291)	1.5% (2/131)	3.8% (2/52)	0% (0/44)	0% (0/35)	0.59	0.11	1.0	1.0
UR^e^	9.6% (28/291)	14.5% (19/131)	13.5% (7/52/)	9.1% (4/44)	22.9% (8/35)	0.18	0.45	1.0	**0.04**
Δ size^f^	0.3 ± 1.0	0.4 ± 1.3	0.3 ± 1.0	0.3 ± 1.9	0.5 ± 0.6	0.58	0.82	0.85	0.40

a P <0.05 is considered significant. The p value of statistical significance was highlighted in bold. P1 Non-carriers vs. all CPGs mutation-carriers, P2 Non-carriers vs. BRCA1 mutation-carriers, P3 Non-carriers vs. BRCA2 mutation-carriers, P4 Non-carriers vs. other CPGs mutation-carriers.

b Mean ± SD, yr, student T test.

c Percentage (No.), Pearson’s chi-square test or Fisher’s exact test.

d The false-negative rate (FNR) was defined as the proportion of the BI-RADS categories less than 4.

e The underestimation rate (UR) was defined as the proportion of the estimated malignancy rate less than 50% (the BI-RADS categories less than 4c).

f The Δ size was calculated by the largest diameter by imaging minus the largest diameter by pathology.

CPG, cancer predisposition genes; MRI, magnetic resonance imaging.

The mammography performed poorly in both the CPG mutation-carriers and non-carriers (FNR=25.4% and 21.3%, *p*=0.29). The UR of the mammography was still higher in evaluating the CPG mutation-carriers than the non-carriers (56.2% *vs*. 44.1%, *p*=6.3×10^-3^), especially in the *BRCA1* mutation-carriers (60.8% *vs*. 44.1%, *p*=6.9×10^-3^; **Table 2** and **Figure S1**). Intriguingly, the ultrasound showed a higher detection rate compared with mammograms regardless of the mutation status (**Table 2**). However, the FNR of ultrasound was significantly higher in patients with GPVs in CPGs than the non-carriers (6.8% *vs*. 2.0%, *p*=2.1×10^-4^; **Table 2** and **Figure S2**), especially in *BRCA1* mutation-carriers (9.1% *vs*. 2.0%, *p*=2.0×10^-4^) and other CPGs mutation-carriers (7.0% *vs*. 2.0%, *p*=0.02). The UR of ultrasound was also higher in patients with GPVs in all CPGs than the non-carriers (35.4% *vs*. 18.7%, *p*=1.5×10^-8^; **Table 2** and **Figure S1**). We also investigated the performance of imaging modalities in patients with mutations in non-*BRCA1/2* cancer predisposition genes which affected no less than 5 patients. As a result, the *RAD51D* mutation carriers showed the highest FNR and UR by ultrasound when comparing with *CHEK2*, *PALB2*, and *TP53* mutation carriers (**Table S1**). The FNRs of MRI were consistently low among different mutation status (0.7% in non-carriers, 1.5% in all CPG mutation-carriers, 3.8% in *BRCA1* mutation-carriers, 0% in *BRCA2* mutation carriers, and 0% in other CPG mutation-carriers); while the UR of MRI was significantly higher in patients with GPVs in CPGs other than *BRCA1/2* than the non-carriers (22.9% *vs*. 9.6%, *p*=0.04; **Table 2** and **Figure S1**). Three modalities showed similar performances measuring the tumor diameters among different mutation status. However, the estimated sizes according to MRI were larger than the tumor sizes (**Table 2**).

### The Accuracy of Combined Imaging Strategies

To evaluate the combined strategies, we also assessed the FNR and UR by combining two imaging techniques. Similar to the performance of ultrasound, the FNR of combining the ultrasound and mammograms was higher in CPG mutation-carriers than the non-carriers (2.8% *vs*. 0.5%, *p*=0.03), especially in *BRCA1* mutation-carriers (5.3% *vs*. 0.5%, *p*=6.7×10^-3^; **Table 3** and **Figure S2**). Its URs were also higher in all CPG mutation-carriers than the non-carriers (27.2% in all CPG mutation-carriers, 27.6% in *BRCA1* mutation-carriers, 25.0% in *BRCA2* mutation carriers, and 30.6% in other CPG mutation-carriers *vs*. 13.8% in non-carriers, *p*=1.6×10^-4^, 5.6×10^-3^, 0.03, and 0.02, respectively; **Table 3** and **Figure S1**). The combination of ultrasound and mammograms performed superior than the ultrasound or the mammograms separately with lower URs in the non-carriers (OR [95%CI] =0.7 [0.5-1.0] and 0.2 [0.1-0.3], *p*=0.04 and 2.3×10^-22^; **Figure S3**). While this combination only showed lower FNR than the mammograms in the non-carriers (OR [95%CI] =0.0 [0.0-0.1], *p*=2.9×10^-26^; **Figure S4**). In CPG mutation-carriers, this combination also showed lower UR (OR [95%CI] =0.3 [0.2-0.5], *p*=2.7×10^-8^; **Figure S3**) and lower FNR (OR [95%CI] =0.1 [0.0-0.2], *p*=9.0×10^-11^; **Figure S4**) than the mammograms. However, the combination of ultrasound and mammograms still showed significantly higher URs than the MRI (OR [95%CI] =2.2 [1.2-4.2], *p*= 8.2×10^-3^; **Figure S3**).

**Table 3:** The performance of combined imaging modalities in patients with cancer predisposition gene mutations and pair non-mutation controls.

Combined imaging accuracy^a^	Paired non-CPG controls	All CPG mutation-carriers	*BRCA1* mutation-carriers	*BRCA2* mutation-carriers	Other CPG mutation-carriers	*P* 1^b^	*P* 2	*P* 3	*P* 4
Ultrasound+ Mammograms									
FNR^c^	0.5% (2/407)	2.8% (5/180)	5.3% (4/76)	0% (0/68)	2.8% (1/36)	**0.03**	**6.7×10^-3^**	1.0	0.23
UR^d^	13.8% (56/407)	27.2% (49/180)	27.6% (21/76)	25.0% (17/68)	30.6% (11/36)	**1.6×10^-4^**	**5.6×10^-3^**	**0.03**	**0.02**
Mammograms+ MRI^a^									
FNR	0.6% (1/180)	2.7% (2/74)	6.3% (2/32)	0% (0/23)	0% (0/19)	0.20	0.06	1.0	1.0
UR	7.8% (14/180)	18.9% (14/74)	18.8% (6/32)	13.0% (3/23)	26.3% (5/19)	**0.02**	0.09	0.42	**0.02**
Ultrasound+ MRI^a^									
FNR	0% (0/278)	1.6% (2/127)	4.1% (2/49)	0% (0/43)	0% (0/35)	0.10	**0.02**	**-**	–
UR	5.4% (15/278)	11.0% (14/127)	12.2% (6/49)	7.0% (3/43)	14.3% (5/35)	0.06	0.11	0.72	0.06

a Percentage (No.), Pearson’s chi-square test or Fisher’s exact test.

b P <0.05 is considered significant. The p value of statistical significance was highlighted in bold. P1 Non-carriers vs. all CPGs mutation-carriers, P2 Non-carriers vs. BRCA1 mutation-carriers, P3 Non-carriers vs. BRCA2 mutation-carriers, P4 Non-carriers vs. other CPGs mutation-carriers.

c The false-negative rate (FNR) was defined as the proportion of the BI-RADS categories less than 4.

d The underestimation rate (UR) was defined as the proportion of the estimated malignancy rate less than 50% (the BI-RADS categories less than 4c).

CPG, cancer predisposition genes; MRI, magnetic resonance imaging.

With the combination of mammograms and MRI, the FNR showed no difference among different subgroups (*p*>0.05; **Figure S2**), while the UR was higher in CPG mutation-carriers than the non-carriers (18.9% vs. 7.8%, *p*=0.02), especially in other CPG mutation-carriers (26.3% *vs*. 7.8%, *p*=0.02; **Table 3** and **Figure S1**). The combination of mammograms and MRI showed lower URs (OR [95%CI] =0.2 [0.1-0.4] and 0.1 [0.1-0.2], *p*=4.1×10^-8^ and 2.1×10^-20^, respectively; **Figure S3**) and lower FNRs (OR [95%CI] =0.1 [0.0-0.3] and 0.0 [0.0-0.1], *p*=5.6×10^-6^ and 2.5×10^-14^, respectively; **Figure S4**) than mammograms in both CPG mutation-carriers and non-carriers. However, the mammograms didn’t benefit the accuracy of MRI in this combination (*p*>0.05; **Figures S3** and **S4**).

Combining the ultrasound and MRI, the FNR was only found higher in *BRCA1* mutation-carriers than the non-carriers (4.1% *vs*. 0%, *p*=0.02; **Figures S2**) but not in *BRCA2* mutation-carriers and other CPG mutation-carriers, and the URs were consistently low among different subgroups (**Table 3** and **Figures S1**). The combination of ultrasound and MRI showed lower URs than the ultrasound alone in both CPG mutation-carriers and non-carriers (OR [95%CI] =0.2 [0.1-0.4] and 0.3 [0.1-0.4], *p*=7.4×10^-8^ and 3.1×10^-8^, respectively; **Figure S3**). Similarly, the ultrasound also didn’t benefit the accuracy of MRI in this combination (*p*>0.05; **Figures S3** and **S4**).

Furthermore, 174 patients without GPVs and 72 patients with GPVs in CPGs have conducted all three imaging modalities. In patients without GPVs, all the patients can be detected by the combination of these three modalities. However, one patient (0.6%) can only be detected by ultrasound, while none patient can only be detected by mammograms or MRI (**Table S2**). In patients with GPVs in *BRCA1*, two patients (6.5%) were only detected by MRI and two patients (6.5%) cannot be detected by the combination of these three modalities. Intriguingly, both the two undetectable lesions were triple-negative breast cancers which were suspected as fibroadenoma. All three combinations of the two imaging modalities showed a satisfactory detection rate in patients with GPVs in *BRCA2*. In patients with GPVs in other CPGs, one 37-years-old patient (5.3%) with a stop-gained variant in *RAD51D* was only detected by MRI (**Table S2**). One of the three lesions missed by both ultrasound and mammograms was triple-negative breast cancer; while two were ER-positive breast cancers.

### The Characteristics Impact the Diagnostic Sensitivity

To identify the characteristics that might impact the diagnostic sensitivity of different imaging techniques, multivariable logistic regression was conducted. The CPG mutation status, age of onset, lymph nodes status, and tumor size measured by ultrasound significantly impacted the FNR in ultrasound (*p*=8.1×10^-4^, 0.01, 6.7×10^-4^, and 0.03, respectively; **Table S3**). However, the family history or the personal history of breast or ovarian cancer showed no impact on the FNR (*p*=0.89 and 0.41, respectively; **Table S3**). The CPG mutation status and the lymph nodes status also significantly impacted the UR in ultrasound (*p*=2.3×10^-9^ and 5.7×10^-7^; **Table S4**).

For the mammograms, only the tumor size measured by mammograms, rather than CPG mutation status and the family history or the personal history, significantly impacted the FNR (*p*=3.0×10^-6^; **Table S5**). Only the lymph nodes status significantly impacted the UR (*p*=0.04; **Table S6**). The FNR of MRI was not relevant to these characteristics (**Table S7**), while the UR of the MRI was only impacted by the lymph nodes status (*p*=6.5×10^-4^; **Table S8**).

## Discussion

In this study, 343 patients (11.5%) with PGVs in CPGs were identified in a consecutive multicenter cohort of female patients with breast cancer. Compared to patients without CPGs, distinct clinical phenotypes including the onset age, family and personal cancer history, and pathological features have been found in patients with *BRCA1*, *BRCA2*, and other CPGs. The diagnosis accuracies of ultrasound, mammograms, MRI, and the combinations of these modalities were investigated in breast cancer patients with or without CPG mutations by calculating the FNRs and URs. Furthermore, the impacts of each characteristic on the diagnostic performance of different imaging techniques were evaluated.

Although mammography has shown satisfactory detection accuracy in Western countries (23), it demonstrated the highest FNRs and URs regardless of the mutation status in this study (**Table 2**), which might result from the high breast density in Chinese women. It has been reported that most breast cancers detected by ultrasound were not detectable at mammography, even in retrospect (24). Compared with the non-carriers, the *BRCA1* mutation-carriers showed more benign morphologic features in mammograms, which resulted in the highest FNR and UR. The less proportion of DCIS (0%) and the presentation of calcifications (25) in *BRCA1* mutation-carriers might also limit the application of mammograms.

Compared with mammograms, ultrasound has advantages including higher sensitivity in women with dense or small breasts, no radiation exposure, lower cost, and easier access in China (12). For ultrasound, the FNRs were significantly higher in patients with GPVs in CPGs except for *BRCA2*, and the URs were higher in all the CPG mutation-carriers. The BRCA-associated breast cancers were commonly assessed as benign lesions by the ultrasound according to the fibroadenoma-like appearance and morphologic features of round or oval masses with circumscribed margins (25, 26). Meanwhile, aggressive pathologic features in *BRCA1* mutation-carriers, such as the higher proportion of grade III tumors (73.0%), resulted in the rapid tumor growth, which has been also suggested as one of the most important underlying factors contributing to the FNR at imaging test (6). Similarly, the high FNR and UR by ultrasound in *RAD51D* mutation carriers might result from their *BRCA1*-like phenotypes, i.e. higher proportion of triple-negative breast cancer (5/8) and higher Ki67 proliferation fractions (6/8 higher than 30%) in this study.

Although some studies showed the ultrasound was comparable with mammograms among women at high risk of breast cancer, the adjunctive ultrasonography could increase the sensitivity of mammograms (14). Consistent with a previous study (27), the addition of the ultrasound to the mammograms would significantly increase the detection rate and diagnostic accuracy regardless of mutation status (**Figures S3** and **S4**). In the Chinese multi-modality independent screening trial (MIST) (28), the supplementary ultrasound after negative mammography result additionally identified 11.9% breast cancer patients (12, 28). However, the FNR in ultrasound alone demonstrated no significant difference from the combination of ultrasound and mammograms in all the patients; the UR was lower through the combination strategy in breast cancer without GPVs.

The MRI has shown the most sensitivity in detecting breast cancer in CPG mutation-carriers even comparing with the combination of ultrasound and mammograms, which was consistent with the previous studies (10, 29, 30). In CPG mutation-carriers, the MRI detected three breast cancers (4.2%) in which the ultrasound and mammograms were undetectable (**Table S2**). In a Japanese case series, two in five primary breast cancers in patients with *BRCA1/2* mutation were only detectable on MRI in a 48-month breast cancer surveillance program including biannual ultrasonography, annual mammography, and MRI (31). A prospective multicenter MRI screening study in Dutch has demonstrated that the supplemental screening MRI would benefit the early cancer detection and the prognosis in women with *BRCA1/2* mutations after an over 9-year follow-up (32). Additionally, MRI has shown better performance than mammograms and ultrasound in dense breasts (33, 34), which are more common in Asian women (2). Therefore, the CPG mutation-carriers were recommended to undergo the MRI for breast cancer surveillance, which might not be replaced by the combination of the ultrasound and the mammograms.

In combination with MRI, the mammograms or the ultrasound seem to have no added value to the sensitivity in both CPG mutation-carriers and non-carriers in this study. Although mammograms have been proved to add value to MRI in older patients (35), whose benefit was limited in young patients especially in *BRCA1/2* mutation-carriers (36, 37). Thus, mammograms might be omitted in younger women who have undergone MRI. However, the *BRCA2* mutation-carriers have a higher proportion of DCIS, which were sometimes only detected as mammographic calcifications (35). While the supplemental mammograms were only proposed in *BRCA2* mutation-carriers to at least age 40 (38). Additionally, the ultrasound was considered as a supplemental screening tool for MRI in *BRCA1/2* mutation-carriers (39), but it has shown no benefit to the MRI in this study.

In the current clinical practice, fewer than 10% of the CPG mutation-carriers are identified (40), which significantly limited the mutation-based breast cancer surveillance. Thus, we testified the impact of the family history and personal history on the detection sensitivities of the three imaging techniques instead of the mutation status. As a result, the family or personal cancer history showed no impact on the FNRs or URs in all three modalities; while the CPG mutation status significantly impacted the FNR and UR in ultrasound but not in mammograms or MRI. Therefore, the genetic test of CPGs should be performed when ultrasound-based surveillance is conducted.

## Limitation

There were several limitations in this study. First, this was a retrospective case-control study to investigate the detection performance of imaging techniques in Chinese CPG mutation-carriers. Second, only patients with breast cancer were enrolled in this study. Thus, specificity was not evaluated in this study. Third, a limited number of patients underwent all three imaging modalities, especially the MRI, which resulted from the accessibility and waiting period of each technique. Meanwhile, the number of patients with mutations in other CPGs except for *BRCA1/2* was also limited. As there was no long-term follow-up in this study, it cannot evaluate the performance of detecting interval cancers. Therefore, double-blind, long-term, randomized prospective clinical trials involving all imaging modalities are needed to verify the diagnostic accuracy, cost-effectiveness, and long-term survival benefits in the future.

## Conclusion

In summary, the genetic etiology of breast cancers is closely correlated with distinct clinical and pathological phenotypes and imaging manifestations. For Chinese breast cancer patients, the ultrasound showed a higher detection rate than mammograms regardless of the mutation status, while its accuracies were lower in CPG mutation-carriers. MRI presented the highest sensitivity, even higher than the combination of ultrasound and mammograms. Additionally, ultrasound and mammograms would add no significant value to MRI for detecting breast cancer in CPG mutation-carriers. Furthermore, the family or personal cancer history cannot replace the mutation status as the impact factor for the false-negative result and underestimation. Clinicians and radiologists should be aware of the atypical imaging presentation of breast cancer in patients with GPVs in CPGs.

## Data Availability Statement

The original contributions presented in the study are included in the article/**Supplementary Material**. Further inquiries can be directed to the corresponding authors.

## Ethics Statement

The study was reviewed and approved by the ethics committee of the Cancer Hospital and Peking Union Medical College Hospital, both of Chinese Academy of Medical Sciences and Peking Union Medical College, and Huanxing Cancer Hospital. The patients/participants provided their written informed consent to participate in this study.

## Author Contributions

JQL, XinW, and XiaW conceived the study. JQL, XinW, LD, XH, JXL, SH, WW, ZX, ZJ, LQ, GL, JW, YL, MZ, and KF enrolled the patients and collected the data. JQL, LD, PY, and JY conducted the genetic tests. JQL and HZ designed the computational framework and analyzed the data. JW, YM, and XL interpreted the medical images. JQL and XiaW supervised the findings of this study. JQL wrote the manuscript. JQL, XinW, LD, and XH contributed equally to this study. All authors contributed to the article and approved the submitted version.

## Funding

This study was funded in part by the National Natural Science Foundation of China (81802669 to JQL), the CAMS Innovation Fund for Medical Sciences (2020-I2M-C&T-B-068 to JQL), the Beijing Hope Run Special Fund (LC2020B05 to JQL), and the CAMS Initiative Fund for Medical Sciences (2016-I2M-1-001 to XiaW).

## Conflict of Interest

The authors declare that the research was conducted in the absence of any commercial or financial relationships that could be construed as a potential conflict of interest.

## Abbreviations

GPV, Germline pathogenic variant; CPG, Cancer predisposition gene; MRI, Magnetic resonance imaging; NCCN, National Comprehensive Cancer Network; USPSTF, United States Preventive Services Taskforce; IARC, International Agency for Research on Cancer; STROBE, Strengthening the Reporting of Observational Studies in Epidemiology; BI-RADS, Breast Imaging Reporting and Data System; FNR, False-negative rate; UR, Underestimation rate; OR, Odds ratio; CI, Confidence interval; MIST, Multi-modality independent screening trial.
