# Supplementary material for: The Distinct Performances of Ultrasound, Mammograms, and MRI in Detecting Breast Cancer in Patients With Germline Pathogenic Variants in Cancer Predisposition Genes
Source: Front Oncol. 2021 Jul 13;11:710156. doi: 10.3389/fonc.2021.710156 (PMC8316045; doi:10.3389/fonc.2021.710156)
Supplement: Supplementary file 1 [file DataSheet_1.pdf]

Supplementary Materials

Figure S1. The Difference in Underestimation Rate of Each Imaging Modality between Mutation Carriers and Non-carriers. 2

Figure S2. The Difference in False-negative Rate of Each Imaging Modality between Mutation Carriers and Non-carriers. 4

Figure S3. The Difference in Underestimation Rate between the Imaging Modalities and the Combined Strategies in Mutation Carriers and Non-carriers. 6

Figure S4. The Difference in False-negative Rate between the Imaging Modalities and the Combined Strategies in Mutation Carriers and Non-carriers. 8

Table S1. The Performance of Imaging Modalities in Patients with Mutations in non-*BRCA1/2* Cancer Predisposition Genes 9

Table S2. The Performance of the Combined Three Imaging Modalities in Patients with Cancer Predisposition Gene Mutations and Pair Non-mutation Controls 10

Table S3. Parameters in the Multivariable Logistic Regression to Predict the False Negative in Ultrasound 11

Table S4. Parameters in the Multivariable Logistic Regression to Predict the Underestimation in Ultrasound 12

Table S5. Parameters in the Multivariable Logistic Regression to Predict the False Negative in Mammograms 13

Table S6. Parameters in the Multivariable Logistic Regression to Predict the Underestimation in Mammograms 14

Table S7. Parameters in the Multivariable Logistic Regression to Predict the False Negative in Magnetic Resonance Imaging (MRI) 15

Table S8. Parameters in the Multivariable Logistic Regression to Predict the Underestimation in Magnetic Resonance Imaging (MRI) 16

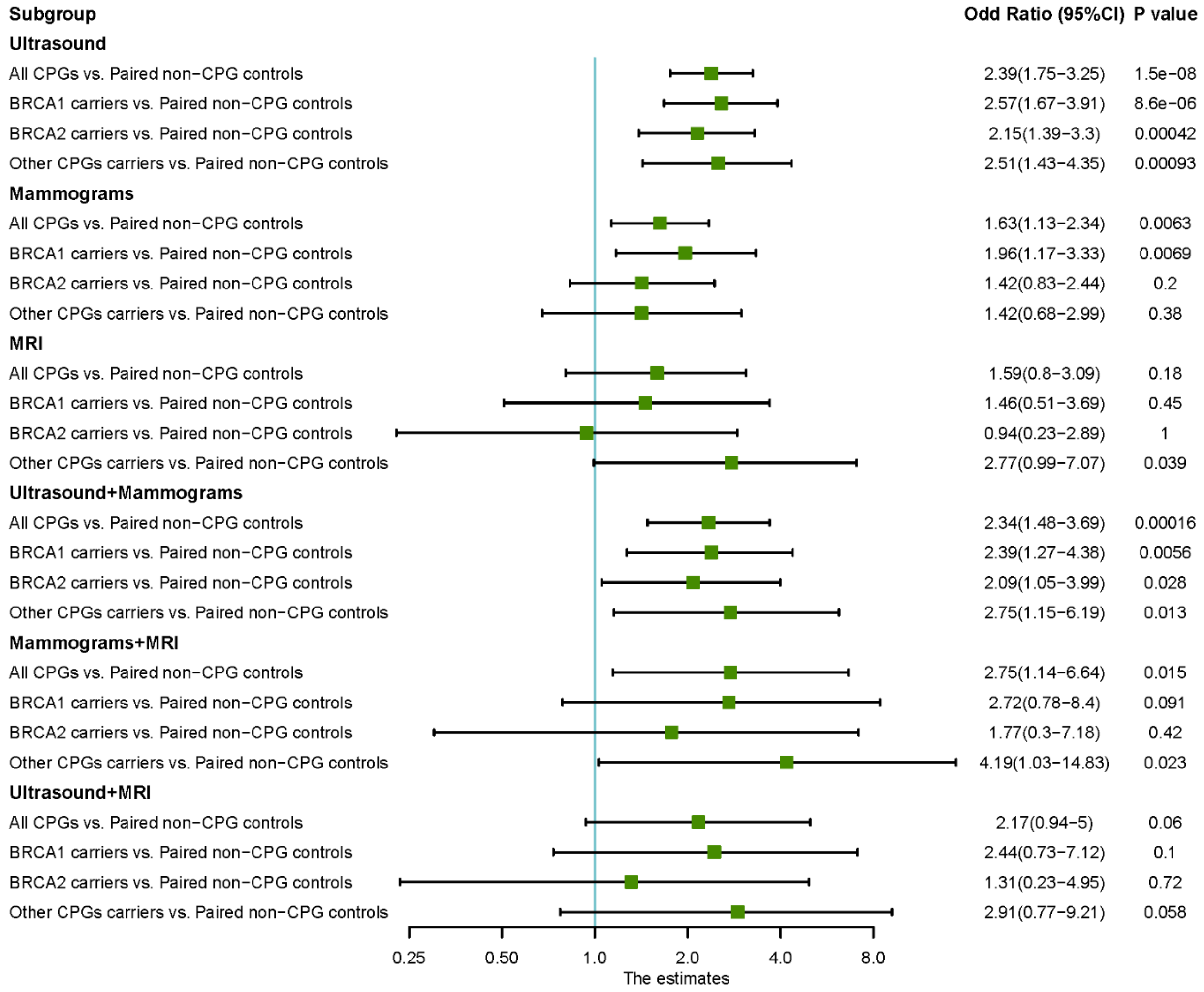

Figure S1. The Difference in Underestimation Rate of Each Imaging Modality between Mutation Carriers and Non-carriers.

The underestimation rate (UR) of the mammography was higher in evaluating the cancer predisposition gene (CPG) mutation-carriers than the non-carriers (56.2% vs. 44.1%, p=6.3×10^{-3}), especially in the *BRCA1* mutation-carriers (60.8% vs. 44.1%, p=6.9×10^{-3}). The UR of ultrasound was also higher in patients with GPVs in all CPGs than the non-carriers (35.4% vs. 18.7%, p=1.5 ×10^{-8}). Also, the UR of MRI was still significantly higher in patients with GPVs in CPGs other

than *BRCA1/2* than the non-carriers (22.9% vs. 9.6%, p=0.04). The URs combining the ultrasound and mammograms were higher in all CPG mutation-carriers than the non-carriers (27.2% in all CPG mutation-carriers, 27.6% in *BRCA1* mutation-carriers, 25.0% in *BRCA2* mutation carriers, and 30.6% in other CPG mutation-carriers vs. 13.8% in non-carriers, $p=1.6\times10^{-4}$, 5.6×10^{-3}, 0.03, and 0.02, respectively). For the combination of mammograms and MRI, the UR was higher in CPG mutation-carriers than the non-carriers (18.9% vs. 7.8%, p=0.02), especially in other CPG mutation-carriers (26.3% vs. 7.8%, p=0.02).

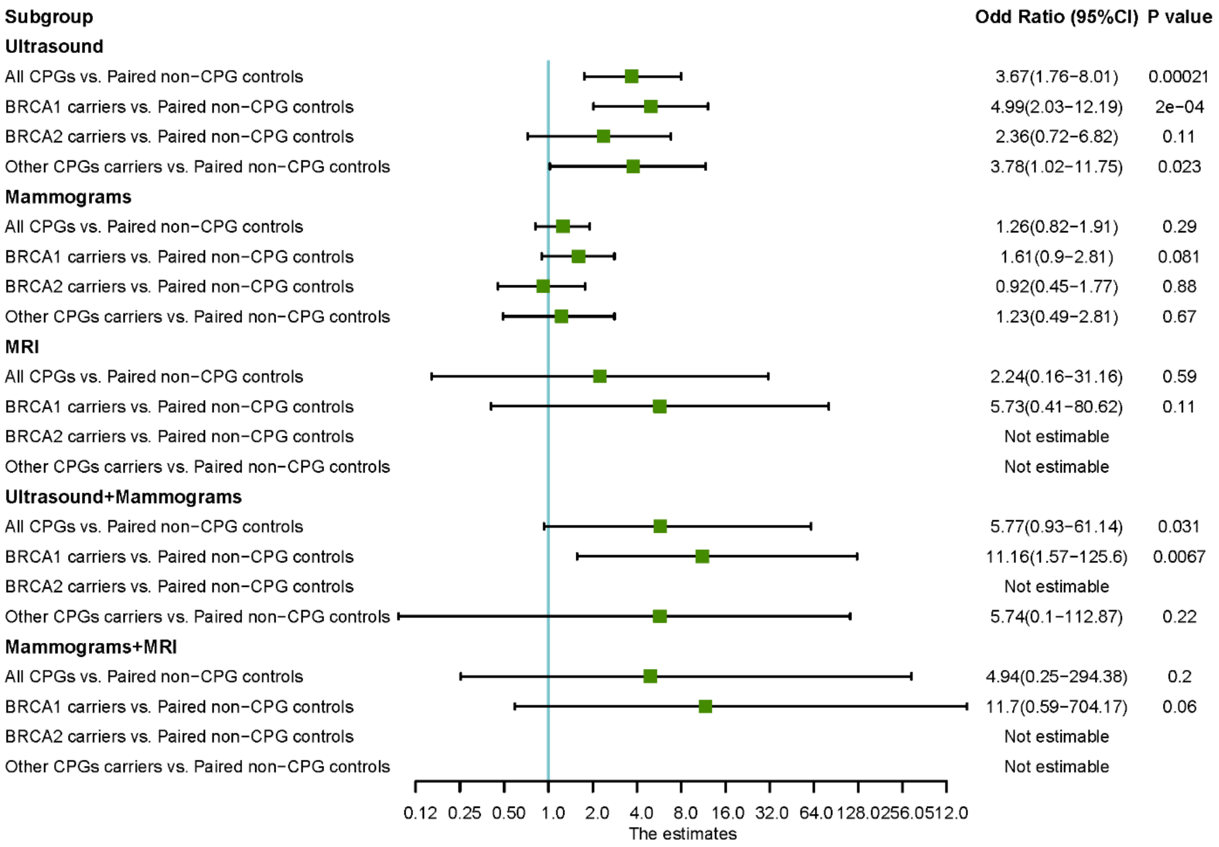

Figure S2. The Difference in False-negative Rate of Each Imaging Modality between Mutation Carriers and Non-carriers.

The false-negative rate (FNR) of ultrasound was significantly higher in patients with germline pathogenic variants (GPVs) in cancer predisposition gene (CPG) than the non-carriers (6.8% vs. 2.0%, p=2.1×10^{-4}), especially in *BRCA1* mutation-carriers (9.1% vs. 2.0%, p=2.0×10^{-4}) and other CPGs mutation-carriers (7.0% vs. 2.0%, p=0.02). However, the FNRs of MRI were consistently low among different mutation statuses (0.7% in non-carriers, 1.5% in all CPG mutation-carriers, 3.8% in *BRCA1* mutation-carriers, 0% in *BRCA2* mutation carriers, and 0% in other CPG mutation-carriers, p>0.05). Similar to the performance of ultrasound, the FNR of combining the ultrasound

and mammograms was higher in CPG mutation-carriers than the non-carriers (2.8% vs. 0.5%, p=0.03), especially in *BRCA1* mutation-carriers (5.3% vs. 0.5%, $p=6.7\times10^{-3}$). For the combination of mammograms and MRI, the FNR showed no difference among different subgroups ($p>0.05$).

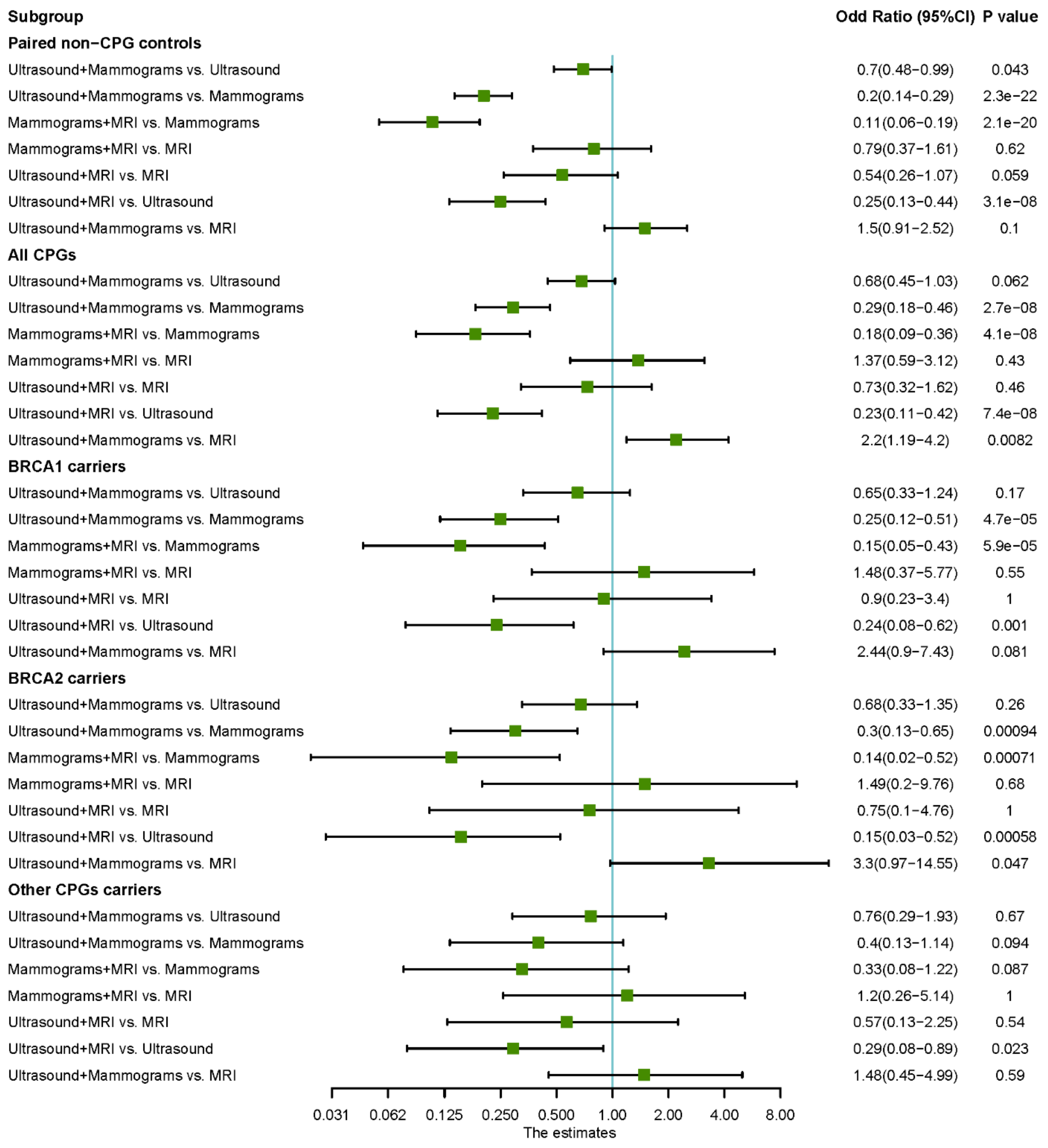

Figure S3. The Difference in Underestimation Rate between the Imaging Modalities and the Combined Strategies in Mutation Carriers and Non-carriers.

The combination of ultrasound and mammograms performed superior than the ultrasound or the

mammograms separately with lower underestimation rates (URs) in the non-carriers (OR [95%CI] =0.7 [0.5-1.0] and 0.2 [0.1-0.3], p=0.04 and 2.3×10^{-22}). In cancer predisposition gene (CPG) mutation-carriers, this combination also showed lower UR (OR [95%CI] =0.3 [0.2-0.5], $p=2.7\times10^{-8}$) than the mammograms. However, the combination of ultrasound and mammograms still showed significantly higher URs than the MRI (OR [95%CI] =2.2 [1.2-4.2], $p= 8.2\times10^{-3}$). The combination of mammograms and MRI showed lower URs than mammograms in both CPG mutation-carriers and non-carriers (OR [95%CI] =0.2 [0.1-0.4] and 0.1 [0.1-0.2], $p=4.1\times10^{-8}$ and 2.1×10^{-20}, respectively). Similarly, the combination of ultrasound and MRI showed lower URs than ultrasound alone in both CPG mutation-carriers and non-carriers (OR [95%CI] =0.2 [0.1-0.4] and 0.3 [0.1-0.4], $p=7.4\times10^{-8}$ and 3.1×10^{-8}, respectively). However, neither the mammograms nor the ultrasound didn't benefit the accuracy of MRI in this combination ($p>0.05$).

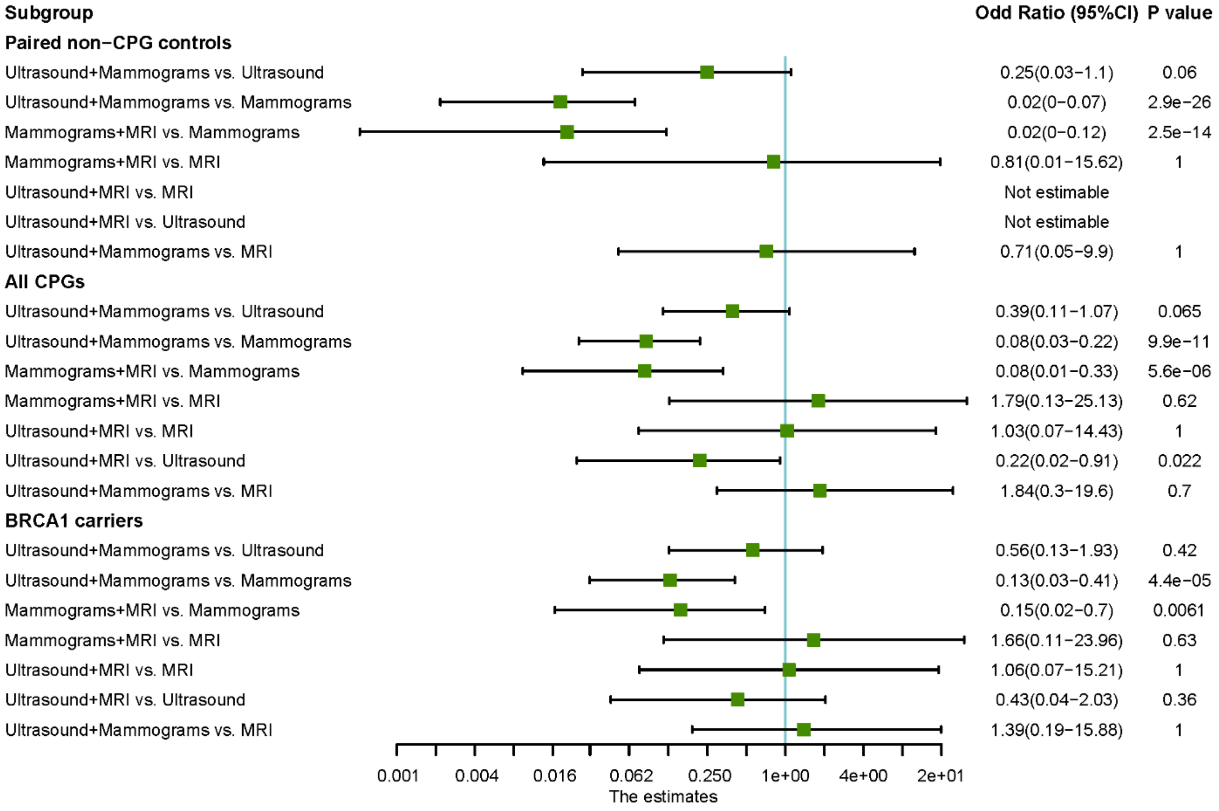

Figure S4. The Difference in False-negative Rate between the Imaging Modalities and the Combined Strategies in Mutation Carriers and Non-carriers.

The combination of ultrasound and mammograms only showed lower false-negative rate (FNR) than the mammograms in the non-carriers (OR [95%CI] =0.0 [0.0-0.1], p=2.9×10^{-26}). In cancer predisposition gene (CPG) mutation-carriers, this combination also showed lower FNR (OR [95%CI] =0.1 [0.0-0.2], p=9.0×10^{-11}) than the mammograms. The combination of mammograms and MRI showed lower FNRs (OR [95%CI] =0.1 [0.0-0.3] and 0.0 [0.0-0.1], p=5.6×10^{-6} and 2.5×10^{-14}, respectively) than mammograms in both CPG mutation-carriers and non-carriers.

Table S1. The Performance of Imaging Modalities in Patients with Mutations in non-*BRCA1/2* Cancer Predisposition Genes

	***PALB2* carriers (n=30)**	***CHEK2* carriers (n=9)**	***RAD51D* carriers (n=8)**	***TP53* carriers (n=5)**
Ultrasound				
FNR[a]	6.67% (2/30)	11.11% (1/9)	12.50% (1/8)	0% (0/5)
UR[b]	30.00% (9/30)	44.44% (4/9)	62.50% (5/8)	20.0% (1/5)
Mammograms				
FNR	33.33% (5/15)	50.00% (1/2)	40.00% (2/5)	0% (0/3)
UR	40.00% (6/15)	50.00% (1/2)	80.00% (4/5)	33.33% (1/3)
MRI				
FNR	0% (0/16)	0% (0/5)	0% (0/3)	0% (0/1)
UR	18.75% (3/16)	20.00% (1/5)	33.33% (1/3)	0% (0/1)

[a] The false-negative rate (FNR) was defined as the proportion of the BI-RADS categories less than 4.

[b] The underestimation rate (UR) was defined as the proportion of the estimated malignancy rate less than 50% (the BI-RADS categories less than 4c).

Abbreviation: MRI, magnetic resonance imaging.

Table S2. The Performance of the Combined Three Imaging Modalities in Patients with Cancer Predisposition Gene Mutations and Pair Non-mutation Controls.

**Detection by imaging modality**	**Paired non-CPG controls (n=174)**	**All CPG mutation-carriers (n=72)**	***BRCA1* mutation-carriers (n=31)**	***BRCA2* mutation-carriers (n=22)**	**Other CPG mutation-carriers (n=19)**
Ultrasound only	0.6% (1)	0% (0)	0% (0)	0% (0)	0% (0)
Mammograms only	0% (0)	0% (0)	0% (0)	0% (0)	0% (0)
MRI only	0% (0)	4.2% (3)	6.5% (2)	0% (0)	5.3% (1)
All negative	0% (0)	2.8% (2)	6.5% (2)	0% (0)	0% (0)

[a] The detection was defined as the proportion of the BI-RADS categories higher than 3.

[d] Percentage (No.)

Abbreviation: CPG, cancer predisposition genes; MRI, magnetic resonance imaging.

Table S3. Parameters in the Multivariable Logistic Regression to Predict the False Negative in Ultrasound

**Variable**	**Regression coefficient**	***P* value**	**OR**	**95% CI**
CPG mutation status	-1.36	**8.1×10^{-4}**	0.26	0-1.05
Age of onset	-0.07	**0.01**	0.93	0.88-0.99
Lymph nodes status	1.14	**6.7×10^{-4}**	3.12	2.30-3.94
Tumor size by ultrasound	-0.40	**0.03**	0.67	0.31-1.03
Family history of breast or ovarian cancer	0.06	0.89	0.94	0.10-1.78
Personal history of breast or ovarian cancer	0.52	0.41	0.60	0-1.81
Intercept	-0.89	0.39	0.41	0-2.45

Abbreviations: OR, odds ratio; CI, confidence interval; CPG, cancer predisposition gene.

P value<0.05 is statistically significant.

Table S4. Parameters in the Multivariable Logistic Regression to Predict the Underestimation in Ultrasound

Variable	Regression coefficient	*P* value	OR	95% CI
CPG mutation status	-1.01	**2.3×10^{-9}**	0.37	0.04-0.70
Age of onset	-0.02	0.13	0.99	0.97-1.00
Lymph nodes status	0.80	**5.7×10^{-7}**	2.22	1.90-2.53
Tumor size by ultrasound	-0.01	0.93	0.99	0.83-1.15
Family history of breast or ovarian cancer	0.33	0.11	1.39	0.99-1.79
Personal history of breast or ovarian cancer	0.28	0.47	1.32	0.56-2.08
Intercept	-0.90	0.12	0.41	0-1.55

Abbreviations: OR, odds ratio; CI, confidence interval; CPG, cancer predisposition gene.

P value<0.05 is statistically significant.

Table S5. Parameters in the Multivariable Logistic Regression to Predict the False Negative in Mammograms

**Variable**	**Regression coefficient**	***P* value**	**OR**	**95% CI**
CPG mutation status	0.27	0.77	1.31	0-3.14
Age of onset	0.001	0.98	1.00	0.91-1.09
Lymph nodes status	1.53	0.08	4.62	2.91-6.32
Tumor size by mammograms	-1.29	**3.0×10^{-6}**	0.28	0-0.81
Family history of breast or ovarian cancer	0.37	0.75	1.44	0-3.72
Personal history of breast or ovarian cancer	18.91	1.00	163719251.36	163703364.78-163735137.94
Intercept	-24.58	1.00	2.1×10^{-11}	0-15886.58

Abbreviations: OR, odds ratio; CI, confidence interval; CPG, cancer predisposition gene.

P value<0.05 is statistically significant.

Table S6. Parameters in the Multivariable Logistic Regression to Predict the Underestimation in Mammograms

**Variable**	**Regression coefficient**	***P* value**	**OR**	**95% CI**
CPG mutation status	-17.88	1.00	1.7×10^{-8}	0-7258.03
Age of onset	-0.01	0.91	1.00	0.91-1.08
Lymph nodes status	-2.24	**0.04**	0.11	0-2.24
Tumor size by mammograms	0.58	0.05	1.78	1.20-2.37
Family history of breast or ovarian cancer	0.70	0.41	2.02	0.34-3.70
Personal history of breast or ovarian cancer	15.47	1.00	1.9×10^{-7}	0-15440.03
Intercept	37.83	1.00	2.7×10^{16}	2.7×10^{16}-2.7×10^{16}

Abbreviations: OR, odds ratio; CI, confidence interval; CPG, cancer predisposition gene.

P value<0.05 is statistically significant.

Table S7. Parameters in the Multivariable Logistic Regression to Predict the False Negative in Magnetic Resonance Imaging (MRI)

Variable	Regression coefficient	*P* value	OR	95% CI
CPG mutation status	-17.32	0.99	3.0×10^{-8}	0-4011.64
Age of onset	0.11	0.60	1.12	0.71-1.52
Lymph nodes status	15.87	0.99	7780534.53	7776112.62-7784956.43
Tumor size by MRI	-1.16	0.62	0.32	0-4.85
Family history of breast or ovarian cancer	15.97	1.00	8640292.30	8633030.46-8647554.13
Personal history of breast or ovarian cancer	14.76	1.00	2568913.63	2551047.01-2586780.24
Intercept	-54.54	1.00	2.1×10^{-24}	0-19786.44

Abbreviations: OR, odds ratio; CI, confidence interval; CPG, cancer predisposition gene.

P value<0.05 is statistically significant.

Table S8. Parameters in the Multivariable Logistic Regression to Predict the Underestimation in Magnetic Resonance Imaging (MRI)

**Variable**	**Regression coefficient**	***P* value**	**OR**	**95% CI**
CPG mutation status	-0.30	0.51	0.74	0-1.62
Age of onset	-0.04	0.13	0.96	0.91-1.01
Lymph nodes status	1.64	**6.5×10^{-4}**	5.18	4.23-6.12
Tumor size by MRI	0.23	0.16	1.26	0.94-1.58
Family history of breast or ovarian cancer	-0.73	0.11	0.48	0-1.37
Personal history of breast or ovarian cancer	19.05	1.00	188099487.01	188077401.71-188121572.31
Intercept	-20.22	1.00	1.7×10^{-9}	0-22085.30

Abbreviations: OR, odds ratio; CI, confidence interval; CPG, cancer predisposition gene.

P value<0.05 is statistically significant.
